# Prevalence of *Blastocystis* in Shelter-Resident and Client-Owned Companion Animals in the US Pacific Northwest

**DOI:** 10.1371/journal.pone.0107496

**Published:** 2014-09-16

**Authors:** Craig G. Ruaux, Bernadette V. Stang

**Affiliations:** Department of Clinical Sciences, College of Veterinary Medicine, Oregon State University, Corvallis, Oregon, United States of America; The University of Hong Kong, Hong Kong

## Abstract

Domestic dogs and cats are commonly infected with a variety of protozoan enteric parasites, including *Blastocystis* spp. In addition, there is growing interest in *Blastocystis* as a potential enteric pathogen, and the possible role of domestic and in-contact animals as reservoirs for human infection. Domestic animals in shelter environments are commonly recognized to be at higher risk for carriage of enteropathogens. The purpose of this study was to determine the frequency of infection of shelter-resident and client-owned domestic dogs and cats with *Blastocystis* spp in the Pacific Northwest region of the USA. Fecal samples were collected from 103 shelter-resident dogs, 105 shelter-resident cats, 51 client-owned dogs and 52 client-owned cats. *Blastocystis* were detected and subtypes assigned using a nested PCR based on small subunit ribosomal DNA sequences. Shelter-resident animals were significantly more likely to test positive for *Blastocystis* (P<0.05 for dogs, P = 0.009 for cats). Sequence analysis indicated that shelter-resident animals were carrying a variety of *Blastocystis* subtypes. No relationship was seen between *Blastocystis* carriage and the presence of gastrointestinal disease signs in either dogs or cats. These data suggest that, as previously reported for other enteric pathogens, shelter-resident companion animals are a higher risk for carriage of *Blastocystis* spp. The lack of relationship between *Blastocystis* carriage and intestinal disease in shelter-resident animals suggests that this organism is unlikely to be a major enteric pathogen in these species.

## Introduction

Companion animals (specifically, domestic dogs and cats) are prone to several protozoan gastrointestinal infections, with *Giardia duodenalis* [syn. *G. lamblia*, *G. intestinalis*] and *Cryptosporidium parvum* both being of significant concern in animals with gastrointestinal disease, and as potential zoonotic infections [1], [2]. Recently, increasing interest has been given to the stramenopile organism *Blastocystis* spp. as a potential cause of gastrointestinal disease in human beings [3], [4]. While the putative link between *Blastocystis* infection and gastrointestinal disease is not without controversy [5]–[7], a growing body of evidence suggests that *Blastocystis* is able to produce cysteine proteinases that mediate interleukin-8 release from enterocytes [8], while also potentially evading recognition by Toll-like receptors [9].

The prevalence of both *Blastocystis* spp. and *Giardia* spp. show marked variations depending upon geographical location and the host species of interest [10]–[12]. In the domestic dog, carriage rates for *Blastocystis* spp. as high as 70% were reported from culture-based studies in shelter-resident dogs in a subtropical environment [13], while more recent studies from essentially the same location found markedly lower prevalence using molecular methods [14].

The purpose of the study described here was to estimate the prevalence of *Blastocystis* spp. in shelter-resident and client-owned companion animals in the US Pacific Northwest region, a geographic region characterized by a moderate, maritime climate. This region has previously been reported to have “hyperendemism” for enteric protozoan infection (specifically, *Giardia* spp.) in a large scale study of fecal samples from dogs submitted for parasitological examination to a commercial laboratory [15]. We hypothesized that shelter-resident dogs and cats would show higher carriage rates for *Blastocystis* than client-owned dogs, and that *Blastocystis* infection would be associated with greater risk of gastrointestinal symptoms in shelter-resident animals.

## Materials and Methods

### Sample collection

Canine and feline stool samples were collected from the Oregon Humane Society facility in Portland, Oregon, and from the Heartland Humane Society facility in Corvallis, Oregon, throughout the summer months of 2012. A minimum of 50 samples from each species of interest (i.e. 50 dogs, 50 cats) were collected from each site, resulting in a total collection of 103 samples from shelter resident dogs and 105 samples from shelter-resident cats. Historical data regarding the stay duration of each animal, breed and estimated age and the presence of gastrointestinal signs while shelter resident were obtained from the computerized records of each shelter and consolidated.

Samples from client-owned control animals were collected from animals belonging to the faculty, staff and students of the College of Veterinary Medicine at Oregon State University and by collection of left over stool samples provided by clients presenting their animals for wellness/vaccination visits at two local veterinary hospitals, one in the south east of the city of Portland, Oregon (VCA South East Portland), and one in the city of Salem (VCA South Salem). This last practice is geographically located approximately halfway between the Corvallis and Portland sites. A minimum of 50 samples from each species of interest was collected, for a total of 103 samples from client owned animals. Data on breed, age, use of anthelmintic medications and the presence of gastrointestinal disease signs in the last 1 and 4 weeks were collected for each sample.

The Oregon State University Institutional Animal Care and Use Committee (IACUC) oversaw all animal use. As this study used only volunteered fecal samples collected after spontaneous defecation, this study was exempt from full IACUC approval. Owners of client-owned animals provided consent for the use of samples from their animals in this study. Collection of samples from the Oregon Humane Society facility in Portland, Oregon, the VCA hospital in South East Portland, Oregon and the VCA hospital in Salem, Oregon was carried out with the full cooperation and knowledge of the medical directors at the respective sites. No endangered or protected species were studied.

Stool samples were stored frozen (−20°C) until further processing.

### Blastocystis control culture

As a source of positive control DNA, *Blastocystis hominis* (Subtype I; American Type Culture Collection, Manassas, VA) were anaerobically cultured in a biphasic medium of inspissated whole egg slants overlaid with 4.5 ml Stone's modification of Locke's solution and 25% heat-inactivated horse serum (ATCC medium 1671), and incubated at 35°C.

### DNA extraction

For positive controls, cultivated *Blastocystis hominis* organisms were isolated from cultures and washed 2 times in fresh Locke's solution. Harvested organisms were added to 0.2 grams of *Blastocystis*-negative dog feces to produce a spiked positive control. DNA was extracted using a commercial DNA extraction kit (QIAmp DNA Stool Mini Kit, Qiagen, Valencia, CA) following the manufacturer's protocol, with DNA extracts eluted in 100 µl of AE buffer. Fecal samples previously tested negative for *Blastocystis* spp were used as negative controls. DNA was stored at −70°C until used. Each DNA extraction batch included a spiked canine fecal sample as a positive control.

For shelter-resident and client-owned canine and feline samples, DNA from 0.20 grams of feces was extracted as described above. The resulting filtrates containing DNA were aliquoted and stored at −70°C until analyzed.

### Amplification and sequencing

The SSU rDNA fragment of *Blastocystis* was amplified using a nested-PCR protocol. Briefly, an approximately 600 base pair fragment of the SSU rDNA was initially amplified using the previously published primers BhRDr and BH1F [1], [16] in a 25 pmol/25 µl reaction using 2 mM MgSO4, 0.2 mM deoxynucleoside triphosphates, 2.5 µg BSA and 1 units of Platinum *Taq* High-Fidelity DNA polymerase (Invitrogen) with 1 µl of DNA template. Reaction cycles consisted of denaturing step of 94°C for 1 minute, and 30 cycles at 94°C for 1 minute, 55°C for 1 minute and 72°C for 1 minute with a final extension cycle at 72°C for 2 minutes. The second amplification was performed using the sense primer Blasto 2F and the anti-sense primer Blasto 2R using PCR conditions previously described [17].

Amplified products were separated by electrophoresis using 1.5% TBE (89 mM Tris-borate, 2 mM EDTA) agarose gel and fragments visualized under UV light after staining with SYBR Safe DNA gel stain (Life Technologies, Grand Island, NY). The DNA fragment size was confirmed using a 100 base pair ladder, selected bands were excised and purified using a commercial kit (QIAquick Gel Extraction Kit, Qiagen, Valencia, CA) according to manufacturer's instructions, and prepared for sequencing. Purified products were sequenced at the Oregon State University Center for Genome Research and Biocomputing. Dilution studies, using spiked whole control organism in a canine-origin fecal sample previously tested negative, indicate that the PCR assay has a sensitivity of 1 organism/200 mg feces (wet weight).

### Subtyping and phylogenetic analysis of blastocystis isolates

Where available, robust and complete sequences from the detected *Blastocystis* organisms were assigned to subtypes using the Blastocystis Sequence Typing website (http://www.publmst.org/blastocystis) [18], [19].

Phylogenetic analyses were conducted using MEGA 5.2.2. Sequences from the *Blastocystis* organisms detected in canine and feline samples were aligned with a collection of sequences for the “barcoding region” [18] of SSU rRNA retrieved from GenBank, with retrieved sequences selected from prior publications[20], [21] to provide representatives of most known subtypes of *Blastocystis* using the subtyping terminology originally proposed by Stensvold and colleagues [22]. Retrieved sequences were aligned using the MUSCLE algorithm [23] as implemented by MEGA 5.2.2, using default parameters. The nucleotide substitution model with the lowest Bayesian information criterion score (Tamura 3-parameter model + discrete Gamma distribution) was selected, and a Maximum-Likelihood phylogenetic tree was calculated using 1500 bootstrap replicates. *Proteromonas lactertae* (AY224080) was used for an out-group.

### Statistical analyses

Data were analyzed using a commercial biological statistics program (GraphPad Prism 5.0 for Macintosh, GraphPad Software, La Jolla, CA). Contingency table analysis (Fischer's Exact Test) was used to assess the frequency of carriage of *Blastocystis* between shelter-resident and client-owned animals, and the frequency of carriage of each parasite between canine and felines. For each host species, additional contingency table analyses were carried out to assess the relative risk of showing signs of gastrointestinal disease during their shelter-resident period (in the case of shelter-resident animals) or during the preceding 1- and 4-week periods in the case of client-owned animals. For all statistical analyses, a calculated value of P<0.05 was assigned for statistical significance.

### Sequence data repositories

Robust sequence data from *Blastocystis* spp. organisms identified in this study were deposited to GenBank.

## Results and Discussion

### Prevalence of Blastocystis spp. infection is higher in shelter-resident than client-owned animals

No *Blastocystis* spp. were detected in any fecal samples from any client-owned animals in this study, with only two very faint bands observed from two separate samples from client-owned animals. These results could not be replicated, and thus these samples were considered either negative or indicative of a very low parasite burden. (The PCR assay has a sensitivity of 1 organism/200 mg feces, see Materials). By comparison, *Blastocystis* organisms were readily detected in 10/103 (9.7%) shelter-resident canines, and 12/103 (11.65%) shelter-resident felines (Table 1). There was no significant difference in *Blastocystis* spp. carriage rates between the shelter-resident dogs and cats (P = 0.686, Fisher's Exact Test). It is interesting to compare the results of this study to previous reports of *Blastocystis* carriage in dogs and cats, and particularly shelter-resident animals. Duda *et al* [13] reported the prevalence of *Blastocystis* in both dogs and cats (70.8% and 67.3%, respectively) from a shelter in southeast Queensland, Australia, a semi-tropical environment, using enrichment culture and light microscopy techniques. In contrast to this, a recent publication using a PCR methodology found only two positive samples from 45 shelter-resident dogs from the same semi-tropical environment [24], and no positive samples from client-owned dogs. Wang *et al* [24] suggest that both methodological differences (light microscopy may be prone to false positive identifications) and differences in hygiene and management of shelter-resident dogs in the intervening years may account for the different prevalence detected. The data reported here indicate a higher prevalence of *Blastocystis* in shelter-resident dogs in the Pacific Northwest of the USA than in the shelter-resident dogs in southeast Queensland, this may be due to differences in management of shelter-resident dogs in the cooler, maritime environment of the Pacific Northwest, or a higher environmental risk of contracting the organism.

**Table 1:** Prevalence of *Blastocystis* spp. infection in shelter-resident dogs and cats in the US Pacific Northwest.

Species	*Blastocystis* Positive	*Blastocystis* Negative
Canine	10	93
Feline	12	93

### Carriage of Blastocystis was not associated with increased risk of gastrointestinal signs in shelter-resident or client-owned dogs and cats

While records of gastrointestinal disease signs, typically diarrhea, were relatively frequent in the medical records of the shelter-resident cats (26/105, 24.7%) and less so in dogs (9/103, 8.7%), there was no significant relationship seen between carriage of *Blastocystis* and the presence of gastrointestinal signs in the shelter-resident animals. Four of 52 (7.7%) client owned cats showed owner-reported gastrointestinal signs in the week prior to collection, while 2/51 (3.9%) of client-owned dogs showed gastrointestinal signs in the week prior to collection. None of the client-owned animals showing gastrointestinal signs tested positive for *Blastocystis*. Shelter-resident cats were significantly more likely to have gastrointestinal disease signs overall than client-owned cats (P = 0.0099, Relative Risk 3.21, Fischer's Exact Test), while there was no significant relationship between shelter-residence and presence of gastrointestinal signs in dogs (P = 0.339, Fischer's Exact Test). Similar data regarding the frequency of diarrhea in shelter-resident dogs and cats have been reported previously [25]. From the data reported in this study, it appears unlikely that the presence of *Blastocystis* infection is a significant contributor to diarrhea in shelter-resident cats in the Pacific Northwest of the USA.

### Shelter-resident dogs and cats carried several different Blastocystis spp. subtypes

**Figure 1:**
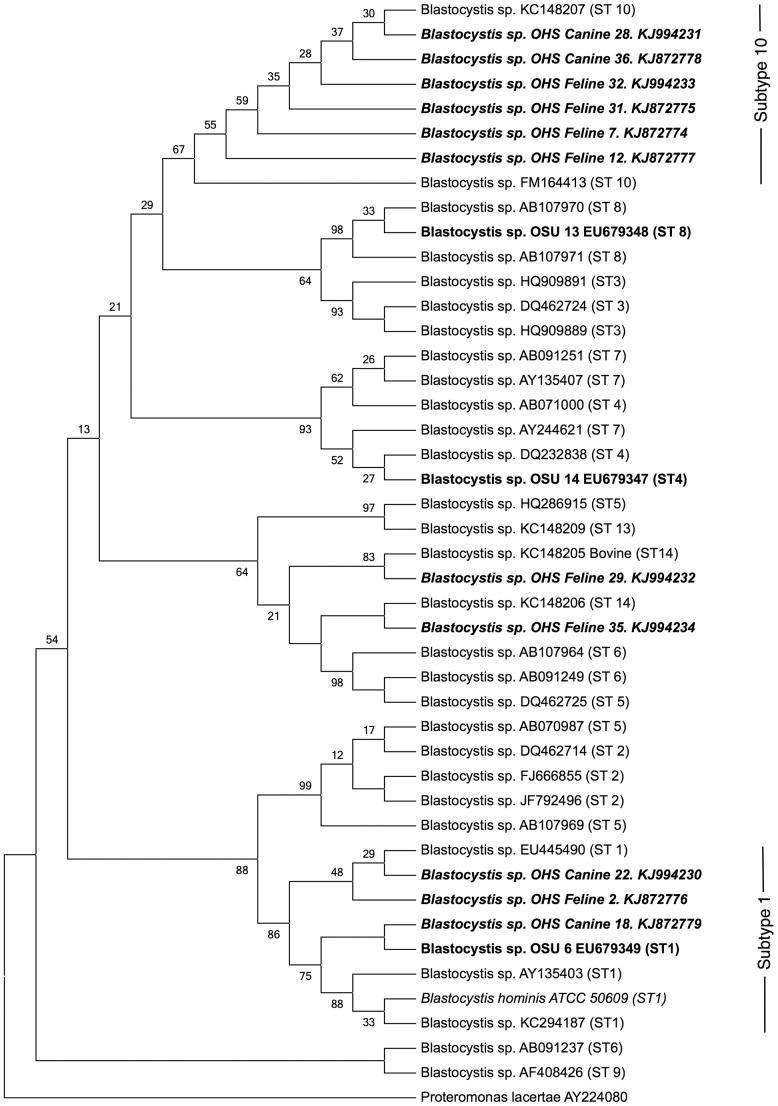
Bootstrap consensus phylogenetic tree for aligned small subunit rDNA sequences from *Blastocystis* spp. isolated from shelter-resident canines and felines, and previously published representative sequences, using *Proteromonas lacerate* as an outgroup. The tree was derived using the Maximum Likelihood method based on a Tamura 3-parameter model, with 1500 bootstrap replicates. Numbers at branch bases indicate the percentage of bootstrap replicates in which the taxa clustered together. Taxa isolated from canine and feline samples are shown in ***bold, italic*** typeface, while organisms isolated from human patients in the same geographical region are shown in **bold** typeface. The positive control organism for the PCR method is shown in *italic*.

Phylogenetic analysis of sequences for SSU rDNA recovered from shelter-resident dogs and cats revealed the presence of several different *Blastocystis* spp. Subtypes (Figure 1, Table 2). Three of the recovered organisms grouped with representative organisms for Subtype 1, using subtype designations previously defined by Stensvold *et al.* [22] The majority of the remaining sequences were identified as subtype 10, a subtype that has previously been identified with highest frequency in artiodactyls, and was not identified in human beings in the same study [26]. One sequence from a shelter-resident feline (KJ994232) was not assigned a type by the sequence-typing database, while the phylogenetic analysis grouped this organism with a subtype 14 strain. A second feline-origin sequence (KJ994234) was assigned to subtype 3 by the sequence-typing database, and again phylogenetic analysis grouped this organism with subtype 14.

**Table 2:** *Blastocystis* spp. subtypes detected in shelter-resident dogs and cats in the US Pacific Northwest.

Genbank ID	Host Species	Subtype
KJ872774	Feline	10
KJ872775	Feline	10
KJ872776	Feline	1
KJ872777	Feline	10
KJ872778	Canine	10
KJ872779	Canine	1
KJ994230	Canine	1
KJ994231	Canine	10
KJ994232	Feline	Unclear, most related to 14
KJ994233	Feline	10
KJ994234	Feline	3

Subtype designations assigned using the *Blastocystis* Sequence Typing website (http://www.publmst.org/blastocystis) [18], [19].

Interestingly, one strain identified in this study, OHS Canine 18 (Subtype 1), segregated with an organism previously identified in a human being within the Willamette valley (OSU 6 EU679349), the location of the study reported here [16]. The human patient from whom this strain was isolated was reportedly demonstrating signs of gastrointestinal disease, while no gastrointestinal signs were noted in the dog from which this strain was isolated. Other workers have reported high rates of *Blastocystis* carriage in domestic animals associated with human beings symptomatic for Blastocystosis [27], however later evidence regarding *Blastocystis* diversity in varying geographic settings suggests that dogs are transient hosts of subtypes with potential for human pathogenicity [24], and thus that dogs do not represent a significant reservoir species for infection of humans. Given that the majority of identifiable subtypes in the study reported here were subtype 10, a subtype with little evidence of carriage in human beings, we feel that it is unlikely that shelter resident animals in the Pacific Northwest of the USA represent a potential source of zoonotic infection of animal handlers or owners.
